# Developmental Analysis of Spliceosomal snRNA Isoform Expression

**DOI:** 10.1534/g3.114.015735

**Published:** 2014-11-21

**Authors:** Zhipeng Lu, A. Gregory Matera

**Affiliations:** * Department of Biology, University of North Carolina, Chapel Hill, North Carolina 27599-3280; † Department of Genetics, University of North Carolina, Chapel Hill, North Carolina 27599-3280; ‡ Integrative Program for Biological and Genome Sciences, University of North Carolina, Chapel Hill, North Carolina 27599-3280

**Keywords:** snRNA isoforms, developmental biology, evolution, RNA-seq, snRNP biogenesis

## Abstract

Pre-mRNA splicing is a critical step in eukaryotic gene expression that contributes to proteomic, cellular, and developmental complexity. Small nuclear (sn)RNAs are core spliceosomal components; however, the extent to which differential expression of snRNA isoforms regulates splicing is completely unknown. This is partly due to difficulties in the accurate analysis of the spatial and temporal expression patterns of snRNAs. Here, we use high-throughput RNA-sequencing (RNA-seq) data to profile expression of four major snRNAs throughout *Drosophila* development. This analysis shows that individual isoforms of each snRNA have distinct expression patterns in the embryo, larva, and pharate adult stages. Expression of these isoforms is more heterogeneous during embryogenesis; as development progresses, a single isoform from each snRNA subtype gradually dominates expression. Despite the lack of stable snRNA orthologous groups during evolution, this developmental switching of snRNA isoforms also occurs in distantly related vertebrate species, such as *Xenopus*, mouse, and human. Our results indicate that expression of snRNA isoforms is regulated and lays the foundation for functional studies of individual snRNA isoforms.

Removal of introns from pre-mRNAs, a process called splicing, is an important step in the expression of eukaryotic genes. Splicing adds an important layer to the spatial and temporal regulation of gene expression, which is essential for the generation of diverse cell types from an identical genome (Chen and Manley 2009). Splicing of most introns is catalyzed by the spliceosome, a macromolecular complex containing five small nuclear ribonucleoproteins (snRNPs) and numerous auxiliary proteins (Will and Luhrmann 2011; Matera and Wang 2014). Two types of spliceosomes coexist in most eukaryotic cells, the major (U2-type) containing U1, U2, U4, U5, and U6 snRNPs and the minor (U12-type) containing U11, U12, U4atac, U5, and U6atac snRNPs. The major spliceosome catalyzes removal of more than 99% of all introns, whereas the minor spliceosome splices less than 1% of introns (Alioto 2007).

The potential for snRNA paralogs to regulate splicing has been recognized since the early 1980s after the discovery of heterogeneity among snRNA populations (Mattaj and Hamm 1989). As a result, a number of studies have analyzed the expression of snRNA isoforms in a variety of organisms (Forbes *et al.* 1984; Lund *et al.* 1985, 1987, 1988; Lund and Dahlberg 1987; Korf *et al.* 1988; Lobo *et al.* 1988; Nash *et al.* 1989; Santiago and Marzluff 1989; Lo and Mount 1990; Hanley and Schuler 1991; Stefanovic *et al.* 1991; Sontheimer and Steitz 1992; Sierra-Montes *et al.* 2002, 2003, 2005; Pereira-Simon *et al.* 2004; Chen *et al.* 2005; Hinas *et al.* 2006; Smail *et al.* 2006; Jia *et al.* 2012; Praveen *et al.* 2012; O’Reilly *et al.* 2013). These studies suggest that certain snRNA isoforms are differentially expressed in various tissues or over developmental time.

The contribution of snRNA variants to the regulation of splicing is unclear. First, due to the lack of a genetically tractable system, early studies were unable to interrogate their biological relevance. Second, sequence analysis of snRNA paralogs across evolution suggests that all multi-copy snRNA genes have undergone concerted evolution, *i.e*., members of a given snRNA gene family are more similar within a species than between species (Pavelitz *et al.* 1995, 1999; Mount *et al.* 2007; Marz *et al.* 2008). Because stable orthologous gene groups do not persist over evolutionary time (groups are usually only detectable within a genus), the possibility of significant functional divergence remains in question.

Most vertebrate snRNAs exist in gene families consisting of dozens of nearly identical copies; therefore, a reverse genetic approach to establish genotype–phenotype correlations for all the snRNA gene copies has not been feasible. Compared with vertebrates, *Drosophila* has a much smaller number of snRNA paralogs: five U1 genes, six U2, three U4, seven U5, and three U6. The other spliceosomal snRNAs are all expressed from single copy genes. The extensive genetic toolkit available for *Drosophila*, in addition to the reduced snRNA copy number make it an ideal system for the analysis of multi-copy snRNA genes.

Massively parallel transcriptome sequencing (RNA-seq) makes it possible to analyze transcripts with high accuracy and nucleotide resolution; therefore, it is well-suited for the analysis of highly similar snRNA paralogs. However, most RNA-seq datasets published thus far, including large-scale projects like modENCODE, have been size selected to exclude abundant medium-size (75 to 300 nt) noncoding (nc)RNAs, such as the spliceosomal snRNAs. To analyze the expression of snRNAs, we identified available mouse and *Drosophila* RNA-seq datasets that contain snRNA reads (see *Materials and Methods*) and performed additional RNA-seq experiments on rRNA-depleted *Drosophila* samples. Using these datasets, we performed a comprehensive analysis of the expression of snRNA paralogs throughout *Drosophila* development, as well as from a few mouse tissues. The results show that snRNA paralogs are differentially expressed during development. The expression patterns are similar in many other distantly related species, despite the lack of conservation in orthologous groups of snRNA genes. These data suggest that the developmental regulation of snRNA isoforms plays an important role in eukaryotic gene expression.

## Materials and Methods

### RNA-seq data files

The following previously published RNA-seq data files used in this study were downloaded from modENCODE, NCBI, and EMBL-EBI. Fly ovaries RIP-seq: GSE35842 (GSM876115 to GSM876134 and GSM1149490 to GSM1149493) (Lu *et al.* 2014). The fly RIP-seq was performed on *Oregon R* (wild-type) and flies with VFP-Sm transgenes. Fly embryos: 12 datasets covering 0–2 hr to 22–24 hr embryo transcriptomes (modENCODE_4607 to modENCODE_4618) (Graveley *et al.* 2011). The fly embryo RNA-seq was performed on fly strains as described on the modENCODE website. S2 cells: GSE32120 (six datasets from control RNAi, SRR345578, SRR345579, and SRR345588-SRR345591) (Smith *et al.* 2011). Fly L3 larvae: two datasets of wild-type (*Oregon R*) early 3^rd^ instar larvae (E. L. Garcia and A. G. Matera, unpublished data). Fly pharate adults (wild-type *Oregon R*): GSE50711(Lu and Matera 2014). Mouse ES cells (from S129 mouse): SRR407407 (Liu *et al.* 2011). Mouse testis (from BALB/c mouse): SRR407405 and SRR407406 (Liu *et al.* 2011). Mouse cerebrum (from BALB/c mouse): SRR018013 and SRR018014 (Liu *et al.* 2011). Mouse fetal head (from FVB/N mouse): GSM566796-GSM566798, GSM566803-GSM566805, GSM566809-GSM566811, and GSM718983 (Huang *et al.* 2011). Mouse CCE differentiated ES cells (from 129/ScEv mouse): GSM566792-GSM566795, GSM566799-GSM566802, GSM566806-GSM566808, and GSM718982 (Huang *et al.* 2011).

### Conversion of formats

Conversion of scarf format to fastq format was performed using fq_all2std.pl with modifications on Phred score conversion, where fq_all2std.pl was originally from the MAQ package (Li *et al.* 2008). Conversion of Phred encoding is performed using EMBOSS seqret (Rice *et al.* 2000).

### Extraction of uniquely mappable reads

See Supplementary Methods for detailed mapping procedure. RNA-seq reads were mapped to the curated snRNA genes using bowtie, allowing no mismatches. Uniquely mappable reads were identified from U1, U4, and U5 snRNAs. Fractions of U2 paralogs were determined by a set of linear equations. Because the variance is bigger in the ovary RIP-seq datasets (due to the inherent variability of the lengthy IP procedure), all RIP-seq datasets were added up to calculate the fraction of reads for each snRNA paralog. The 12 embryo RNA-seq datasets were divided into three stages: early (0–8 hr), mid (8–16 hr), and late (16–24 hr) (Graveley *et al.* 2011). This is because we did not see significant variation in the fractions at each embryonic stage. The estimated time intervals are as follows. Ovary to early embryo: 1–2 days (later-stage egg chambers contribute more to the total sequenced snRNAs). Early to middle embryo: 8 hr. Middle to late embryo: 8 hr. S2 cells were derived from late embryos (20–24 hr after egg laying), and therefore later than late embryos. Late embryo to 3^rd^ instar larva: 50 hr. Third instar larva to pharate adult: 140 hr. Note that the intervals among the developmental stages are not constant.

SDs of the fractions were calculated for each stage shown in Figure 3 provided that more than one sample was available. Sample numbers for each stage are as follows. For *Drosophila* snRNAs: ovary, n = 1; early_emb, n = 4; mid_emb, n = 4; late_emb, n = 4; S2, n = 6; L3, n = 2; and pharate, n = 4. For mouse snRNAs: testes, n = 2; and cerebrum, n = 2. Note that each of the *Drosophila* samples typically comprised 30 to 50 animals, whereas the mouse samples were from derived individual animals.

To identify reads from potential new variants, we mapped all the Illumina RNA-seq reads using bowtie2 with default parameters (very-fast option) and calculated the percentage of reads that contained mismatches.

## Results and Discussion

### Generation and identification of appropriate RNA-seq datasets

To analyze the expression of *Drosophila* snRNA paralogs, we first collected published RNA-seq data that contain snRNAs (Table 1). In a previous study, we performed RNA-immunoprecipitation sequencing (RIP-seq) on *Drosophila* Sm proteins on ovarian lysates and these data were used to quantify snRNA levels in ovaries (Lu *et al.* 2014). The snRNAs not bound by Sm proteins are unstable; therefore, the snRNAs recovered from Sm protein IPs accurately reflect the snRNA population (Sauterer *et al.* 1988; Praveen *et al.* 2012). Similarly, snRNA measurements from RNA-seq also reflect the number of functional snRNPs *in vivo*. The fruitfly ovary contains a mixture of somatic and germline cells. Because eggs provide most of the cellular material for early embryogenesis, for the purpose of developmental analysis, we consider the ovary as a developmental stage that is prior to the embryo. We searched public databases and found two additional RNA-seq datasets that contain snRNAs, and these data came from embryos and S2 cells (Graveley *et al.* 2011; Smith *et al.* 2011). S2 cells are derived from 20- to 24-hr late-stage embryos (Schneider 1972); therefore, we compared them to late-stage embryos in our subsequent analysis. In addition, we performed RNA-seq on rRNA-depleted total RNA samples from early 3^rd^ instar larvae and pharate adults (Garcia *et al.* 2013; Lu and Matera 2014). In summary, our data collection covers the major stages of *Drosophila* development: pre-embryo, embryo, larva, and pharate adult (Table 1).

**Table 1:** RNA-seq datasets used in this study

Source	Platform	Samples	Length	Experiment	Reference
*Drosophila* ovaries	Illumina	24	35	RIP-seq	Lu *et al.* 2014
*Drosophila* embryos 0–24 hr	SOLiD	12	50	Ribo(−)	Graveley *et al.* 2011
*Drosophila* S2 cells	Illumina	6	45, 50	Ribo(−)	Smith *et al.* 2011
*Drosophila* 3^rd^ instar larvae	Illumina	2	48	Ribo(−)	Garcia *et al.*, unpublished data
*Drosophila* pharate adults	Illumina	4	48	Ribo(−)	Lu and Matera 2014
Mouse ES cells	SOLiD	1	48	Ribo(−)	Liu *et al.* 2011
Mouse differentiated ES cells	Illumina	12	51	Ribo(−)	Huang *et al.* 2011
Mouse fetal head	Illumina	10	51	Ribo(−)	Huang *et al.* 2011
Mouse cerebrum	SOLiD	2	33	Ribo(−)	Liu *et al.* 2011
Mouse testis	SOLiD	2	33	Ribo(−)	Liu *et al.* 2011

Ribo(−) indicates an RNA-seq experiment in which the ribosomal RNAs are depleted from the total RNA samples.

For evolutionary comparisons, we compiled RNA-seq data containing mouse snRNAs from several types of cells and tissues (Table 1), including embryonic stem (ES) cells, differentiated ES cells, fetal head, cerebrum, and testis (Cui *et al.* 2010; Yang *et al.* 2011). Despite the fact that these samples are not derived from a single lineage, they represent the full range of development, from undifferentiated to terminally differentiated cells. These data were used in comparison with the analysis of fruitfly snRNAs.

### Structural and functional alignment of snRNA isoforms

**Figure 1:**
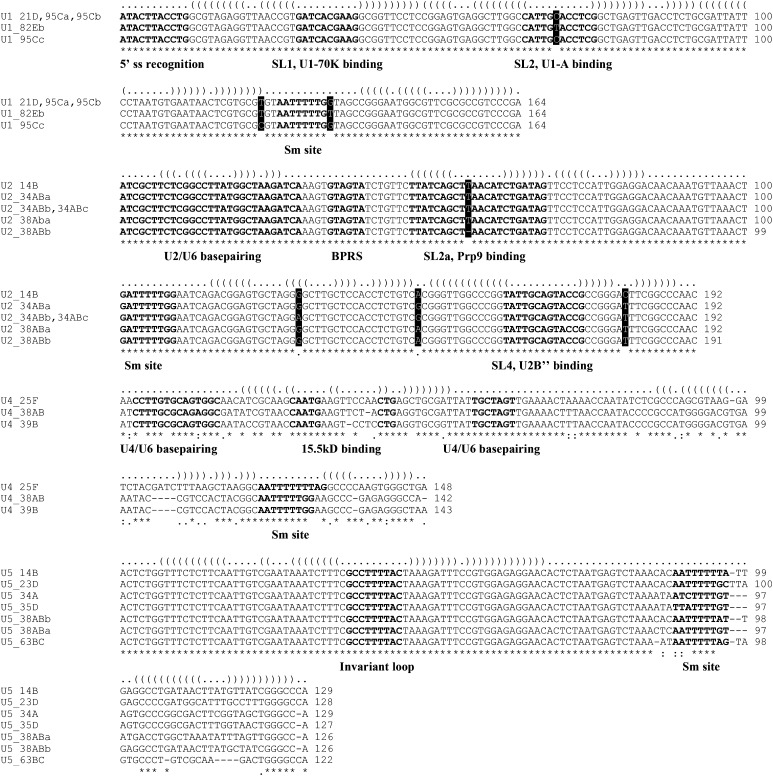
Alignment of *Drosophila* snRNA paralogs. The secondary structure of each snRNA is presented on the top line of each alignment using the dot-bracket notation. The U1 and U2 paralogs have very few variable nucleotide positions (three for U1 and four for U2), and they are highlighted with the black background and white lettering. Sequence elements that are important for base-pairing with other RNAs or interaction with proteins are indicated. U1:21D, U1:95Ca, and U1:95Cb are identical. U2:34ABb and U2:34ABc are identical. 5′ ss recognition: sequence recognizing pre-mRNA 5′ splice site. BPRS: branch-point recognition sequence. SL1, SL2, SL2a, and SL4: stem loops. U4 and U5 paralogs have significant differences among them and U5 paralogs are the most diverse. The 3′ stem loop secondary structure of U5 isoforms is conserved, despite the divergence on the sequence level. Reads covering U4:25F (nucleotides 1–47), U4:38AB (1–46), and U4:39B (1–46) are unique among the three U4 paralogs. Reads covering U5:63BC (96–122) and the other six (97–end) are unique among all U5 paralogs. See Supplementary Methods for details of read mapping.

**Figure 2:**
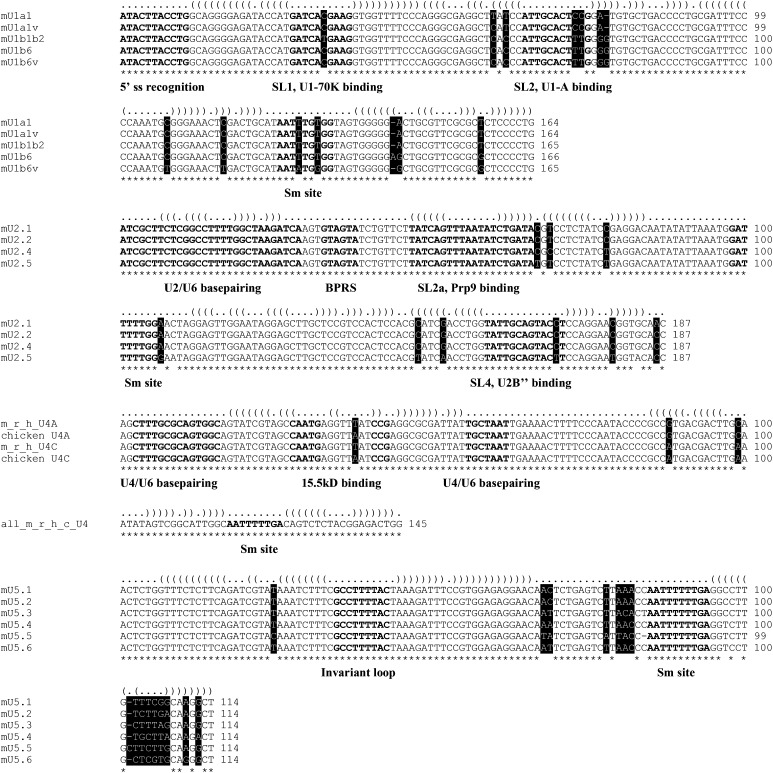
Alignment of mouse snRNA paralogs. Nucleotide variations are highlighted with the black background and white letters. See Figure 1 for abbreviated motif names. Sequence elements that are important for base-pairing with other RNA subtypes or interaction with proteins are indicated. Mouse (m), rat (r), chicken (c), and human (h) U4 snRNA paralogs are aligned together to show the two orthologous groups. Interestingly, even though the U4 snRNAs in several vertebrate species, human, mouse, rat, and chicken, have only three nucleotide variations, they clearly segregate into two groups, based on the two variants in the second stem-loop. Similar to fly U5 snRNAs, mouse U5 paralogs are also the most diverse, and the variable region is confined to the 3′ end. See Supplementary Methods for details of read mapping.

The *Drosophila* genome encodes 27 spliceosomal snRNA genes that belong to nine different snRNA subtypes. The five major spliceosomal snRNAs are each expressed from multiple genes and (with the exception of U6) have multiple nucleotide differences. Generic RNA-seq read mappers, *e.g.*, Bowtie, randomly assign individual reads to multiple mappable locations in the genome; therefore, the measurements of snRNA isoform expression levels are not accurate. To overcome this problem, we aligned U1, U2, U4, and U5 snRNA paralogs and identified variable nucleotides and regions (Figure 1). Due to the repeated nature of the *bona fide* snRNA gene loci as well as the presence of pseudogenes and other repetitive elements nearby (Denison *et al.* 1981; Matera *et al.* 1990; Domitrovich and Kunkel 2003), the human, mouse, and other vertebrate genome assemblies do not accurately reflect the organization of snRNA genes. To analyze the expression of mouse snRNA isoforms, we retrieved known snRNA sequences and performed BLAST searches against genome sequence databases. Available mouse snRNA isoforms were aligned in a manner similar to their fly counterparts (Figure 2).

To help understand how differential expression of snRNA isoforms affects their functions *in vivo*, we superimposed the alignments of snRNA paralogs with the sequence elements known to be required for interaction with proteins and base pairing with other RNA molecules (Figure 1 and Figure 2) (Madhani and Guthrie 1992; Nagai *et al.* 2001; Will and Luhrmann 2011; Lin and Xu 2012). Note that certain nucleotide variations overlap with important sequence and structure motifs and are likely to affect the functions of these isoforms.

### Developmental changes in *Drosophila* snRNA isoform dominance

**Figure 3:**
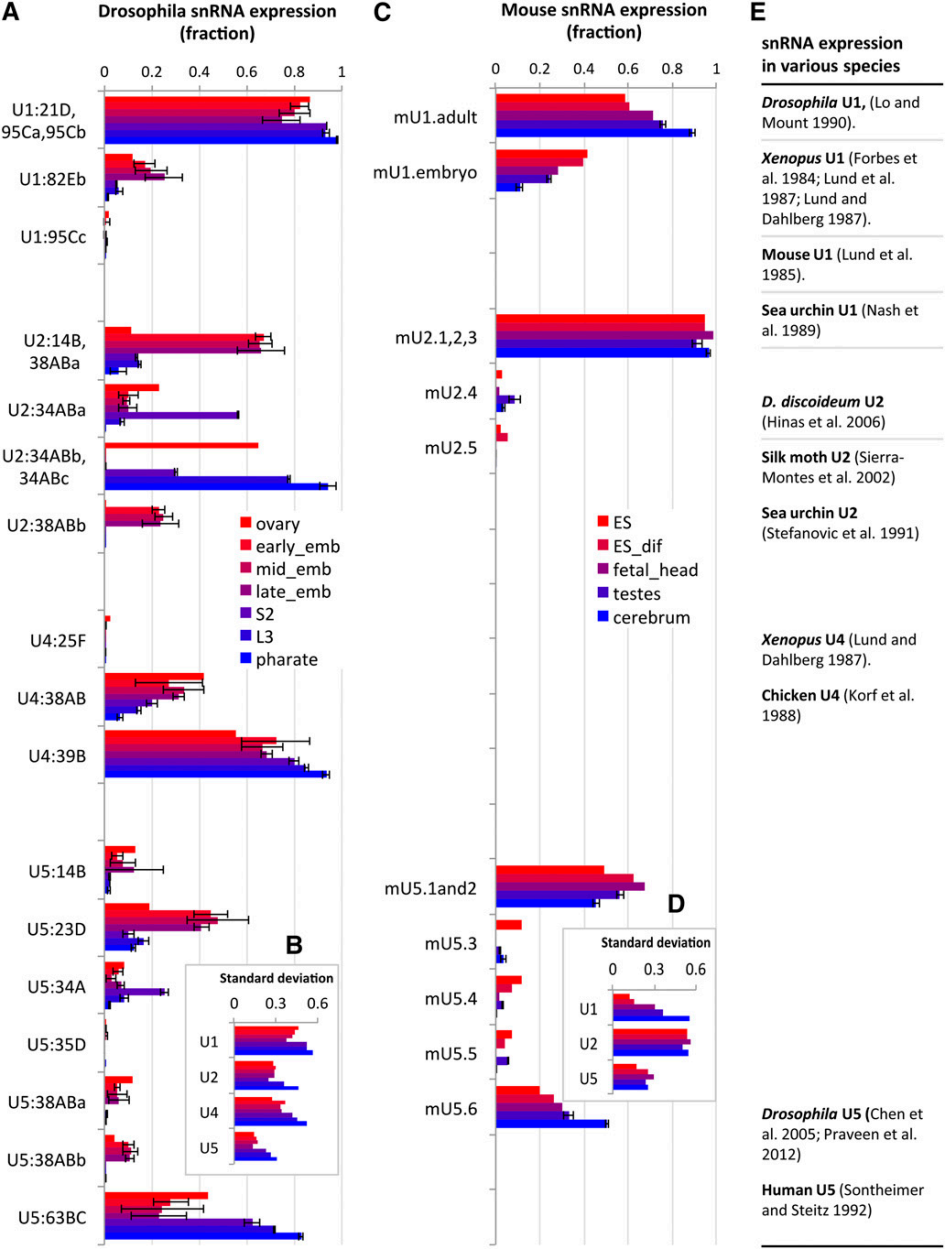
Differential expression of snRNA paralogs during development. (A and C) The fractional expression level for each snRNA paralog was calculated from reads mapping to the variable regions shown in Figure 1 and Figure 2. The fractions for the paralogs of each snRNA subtype add up to 1 in each stage. (A) U2:14B and U2:38ABa are not identical, but they are lumped together due to an insufficiency in read numbers for embryos. (B and D) The SD of the fractional expression values for each group of snRNA isoforms was calculated for each developmental stage. See *Materials and Methods* for the number of samples in each stage. (E) Summary of previous studies on snRNA isoform expression patterns in various species.

To determine the relative expression of each snRNA isoform, we extracted unique sequencing reads mapped to variable regions based on the sequence alignments of fly and mouse snRNAs (Figure 1 and Figure 2). For each RNA-seq experiment (*e.g.*, a certain developmental stage or a cell/tissue type), we calculated the fraction of reads that each isoform uses in each snRNA group (see Supplementary Methods for details of mapping unique reads to snRNA isoforms). This analysis showed that snRNAs that express multiple isoforms exhibit a developmental switch from expressing multiple isoforms during early stages to expressing a single dominant isoform in adults (Figure 3, A and B). Given the mobile nature of snRNA genes, we tested the possibility that additional variant snRNA genes might be expressed. Our examination of sequencing reads mapped to known snRNA loci revealed very few additional reads with mismatches (Supporting Information, Table S1), suggesting that even if other variants exist, their expression is low and does not affect the calculated relative expression level of known snRNA isoforms.

#### U1 snRNA:

Five U1 snRNA genes exist in *Drosophila*, and they express three isoforms, U1:21D/95Ca/95Cb, U1:82Eb, and U1:95Cc. In all the stages analyzed, the U1:21D/95Ca/95Cb isoform is the dominant one, representing 70–98% of total U1 snRNA (Figure 3A; see Table S2 for numbers of unique raw read mapped to each snRNA isoform). Expression of U1:21D/95Ca/95Cb gradually increases during development to almost 100% in adults, whereas U1:82Eb gradually decreases to barely detectable levels. A previous semi-quantitative analysis showed a similar expression pattern for these three isoforms during fly development (Lo and Mount 1990).

#### U2 snRNA:

The six *Drosophila* U2 snRNA genes express five distinct isoforms (Figure 1). The nucleotide variations allow us to analyze them in four groups because some of the variable regions are close to the snRNA ends and few reads are available to distinguish them. U2:34ABb/34ABc is the major isoform in the ovary, representing more than 60% of total U2 (Figure 3A). Its expression decreased sharply in embryos to barely detectable levels later in development. In embryos, U2:14B and U2:38ABa are the dominant isoforms, representing more than 60% of total U2. In later stages, U2:34ABb/34ABc gradually increased to more than 90% in pharate adults, becoming the dominant isoform. U2:38ABb is expressed only in the embryonic stages and barely detectable in ovary or after embryogenesis. S2 cells mainly express U2:34ABa, which is different from all the other samples. Overall, U2:14B/38ABa and U2:34ABb/34ABc display reciprocal expression trends during fly development.

#### U4 snRNA:

Expression of all three *Drosophila* U4 isoforms can be measured accurately due to their divergence (Figure 1 and Figure 3A). U4:25F is barely detectable in any of the stages analyzed, and it is likely a pseudogene may only be expressed from a small number of cells. The expression levels of the other two isoforms, U4:38AB and U4:39B, are similar to each other in earlier stages, including the ovary and embryo. As development progresses, U4:39B gradually takes over the U4 population, generating more than 90% of the total U4 reads in pharate adults. Consistent with the fact that U4:39B is the major isoform expressed in flies, a *P* element insertion in U4:39B is lethal (Y. Wen and A. G. Matera, unpublished observations).

#### U5 snRNA:

All seven *Drosophila* U5 snRNAs can be clearly distinguished from each other (Figure 1). Similar to U1, U2, and U4 snRNAs, our analysis showed a clear developmental bias in U5 isoform expression (Figure 3A). U5:14B, 34A, 35D, 38ABa, and 38ABb are expressed at very low levels in all the stages analyzed. U5:23D and U5:63BC are expressed at very high levels, and their expression follows a reciprocal pattern. Whereas U5:23D is the dominant isoform in embryos, accounting for more than 40% of total U5, its expression decreases to 15% after embryogenesis. U5:63BC accounts for approximately 30% of total U5 reads in embryos, and its expression increases dramatically during development to more than 80% in pharate adults. These results are consistent with previous semi-quantitative analyses of U5 snRNAs (Chen *et al.* 2005; Praveen *et al.* 2012).

The analysis of U1, U2, U4, and U5 snRNAs in fly RNA-seq data revealed preferential expression of snRNA isoforms. We calculated the SD of the fractional expression values for isoforms within each snRNA subtype at each developmental stage and the results are shown in Figure 3B. The overall trend of increasing SDs over developmental time provides further evidence that the dominance of one isoform in later stages of development is a common feature among different spliceosomal snRNA subtypes.

### Developmental analysis of mouse snRNA isoform dominance

To determine whether the developmental expression pattern of *Drosophila* snRNA isoforms is conserved in vertebrates, we analyzed the expression profiles of mouse snRNAs. In addition, we compared the results with those of other published studies (Figure 3, C–E; see Table S3 for numbers of unique raw reads mapped to mouse snRNA isoforms).

#### U1 snRNA:

Previous studies divided mouse U1 snRNAs into embryonic and adult isoforms, each of which are heterogeneous (Figure 2) (Lund *et al.* 1985). Despite the lack of orthologous groups between mouse and fly, we observed a similar pattern of expression during mouse development, consistent with previous reports (Figure 3, C and E) (Lund *et al.* 1985). Similar to flies, the SD of fractions also showed an increasing trend (Figure 3D). The changes in snRNA isoform dominance are also recapitulated in mouse ES cell differentiation (Cheng *et al.* 1997). Studies in *Xenopus* showed that different U1 snRNA isoforms are expressed in oocytes/embryos compared with adults (Lund *et al.* 1987; Lund and Dahlberg 1987). Studies in sea urchins also suggest that U1 isoforms are developmentally regulated (Nash *et al.* 1989). Taken together, these results reveal a similar expression pattern; the major isoform of U1 snRNA is expressed throughout development, whereas the less abundant isoforms are primarily expressed in early embryos and are turned off as development progresses.

#### U2 snRNA:

Available mouse U2 snRNA reads only allow us to distinguish three groups (Figure 2). We found that mU2.1 and mU2.2 dominate U2 snRNA expression in all tissues analyzed (Figure 3C). This analysis is hampered by the fact that we are unable to distinguish all mouse U2 isoforms. Nevertheless, a recent study showed that a 5 base deletion in a mouse U2 snRNA paralog resulted in a recessive neurodegenerative phenotype (Jia *et al.* 2012). The mutated U2 gene was shown to be expressed primarily in the central nervous system and the mutation reportedly caused tissue-specific splicing defects (Jia *et al.* 2012). Hence, snRNA paralogs are not equivalent and may acquire tissue-specific expression patterns.

Previous analysis of U2 snRNA expression in *D. discoideum* also suggests that one group of U2 isoforms decreases dramatically during development relative to the other group (Hinas *et al.* 2006). Analysis of U2 isoforms in silk moth showed a complex pattern of expression, with distinct isoforms dominating each stage (Sierra-Montes *et al.* 2002). Studies in sea urchins showed that U2 isoforms are developmentally regulated (Stefanovic *et al.* 1991). Despite the complication of more isoforms for U2 snRNA, these results together with our analysis showed that more isoforms are expressed in earlier stages and, as development progresses, one isoform takes over the whole population.

#### U4 snRNA:

We could not analyze the expression pattern of mouse U4 snRNAs due to the low number of mappable reads. However, a very similar switching of U4 isoform expression has been shown during *Xenopus* and chicken development (Lund and Dahlberg 1987; Korf *et al.* 1988).

#### U5 snRNA:

Similar expression changes in U5 isoforms are also observed in mice, although the change is not as dramatic (Figure 3, C and D). The mU5.6 and mU5.1and2 RNAs showed reciprocal expression patterns, whereas the other isoforms are expressed at very low levels. Previous analysis of human U5 snRNAs also revealed developmental isoform switching (Sontheimer and Steitz 1992).

## Conclusions

The major Sm-class snRNAs are typically expressed from multi-copy gene families. Here, we performed a comprehensive analysis of snRNA expression patterns in *D. melanogaster* and compared it with other species. Our analysis showed that, despite the lack of stable orthologous groups, developmental switching of snRNA isoforms was similar between vertebrates and invertebrates. This analysis assumes that the relative snRNA gene numbers are constant between the different samples. Thus, genome rearrangements (*e.g.*, in S2 cells compared with fly lines or between animal strains) could skew the results.

The consistent changes in snRNA isoform dominance underline the functional importance of maintaining several genes for each snRNA subtype. During the early stages of development it is likely that expression from multiple snRNA genes is simply needed to support the higher rates of cellular proliferation. However, individual snRNA paralogs may also exert regulatory effects on splicing. Expression from different snRNA gene copies makes it possible to regulate production of specific snRNPs and, therefore, to influence splicing. Different snRNA isoforms might form structurally distinct snRNPs with divergent functions. Consistent with this idea, Bach *et al.* (1990) reported that mouse U1 snRNA isoforms have distinct affinities for U1 snRNP-specific proteins. In conclusion, the comprehensive analysis of snRNA expression in a genetically tractable system provides essential information to help guide future functional studies on snRNA isoforms *in vivo*.

## Supplementary Materials

Supplementary materials are available for this article. We provide a detailed description of the methods for extracting uniquely mappable reads for snRNA paralogs from RNA-seq data, including command line instructions.

Supporting Information
