# Supplementary material for: Developmental Analysis of Spliceosomal snRNA Isoform Expression
Source: G3 (Bethesda). 2014 Nov 21;5(1):103–10. doi: 10.1534/g3.114.015735 (PMC4291461; doi:10.1534/g3.114.015735)
Supplement: Supporting Information [file supp_5_1_103__index.html]

Developmental Analysis of Spliceosomal snRNA Isoform Expression — Supporting Information 

# Developmental Analysis of Spliceosomal snRNA Isoform Expression

## Supporting Information for Lu and Matera, 2015

**Files in this Data Supplement:**

- Supporting Information - File S1 and Tables S1-S3 (PDF, 230 KB)
- File S1 - Assigning RNA-seq reads to *Drosophila* and mouse snRNA isoforms. (PDF, 201 KB)
- Table S1 - All fly and mouse Illimina RNA-seq reads were mapped to the curated snRNA sequences using bowtie2 (-very-fast, default parameters). The percentage of reads with mismatches were calculated for each snRNA group. (.xlsx, 8 KB)
- Table S2 - Numbers of unique reads mapped to each fly snRNA (U1, U4 and U5) are listed. Unique reads that are mapped to each variant position for U2 are listed. (.xlsx, 12 KB)
- Table S3 - Numbers of unique reads mapped to each mouse snRNA (U2, U4 and U5) are listed. Unique reads that are mapped to each variant position for mouse U1 are listed. (.xlsx, 10 KB)
