# Supplementary material for: Developmental Analysis of Spliceosomal snRNA Isoform Expression
Source: G3 (Bethesda). 2014 Nov 21;5(1):103–10. doi: 10.1534/g3.114.015735 (PMC4291461; doi:10.1534/g3.114.015735)
Supplement: Supporting Information [file supp_g3.114.015735_FileS1.pdf]

File S1

Assigning RNA-seq reads to *Drosophila* and mouse snRNA isoforms

In reading this supplemental methods section, you may find the following related publications useful:

Lu Z., Guan X., Schmidt C.A. and **Matera A.G.** (2014). RIP-seq analysis of eukaryotic Sm proteins identifies three major categories of Sm-containing ribonucleoproteins. *Genome Biology* **15**: R7.
Lu Z. and **Matera A.G.** (2014). Vicinal: a method for the determination of ncRNA ends using chimeric reads from RNA-seq experiments. *Nucleic Acids Research*, **42**: e79.

Drosophila snRNAs

There are a total of 29 genes for all the 11 *Drosophila* Sm class snRNAs (see the table below). Even though there are 33 named snRNAs, 5 of them are mistakes in annotation. U1, U2, U4 and U5 snRNA paralogs have nucleotide variations, which can be used to assign uniquely-mappable reads correctly. The three U6 snRNA paralogs are identical, and therefore it is not possible to analyze each separately. The default setting for Bowtie assign repetitive reads to mapped locations uniformly, and is thus not accurate in determining expression levels. Here we use the variable regions in the snRNA paralogs to reassign the reads. Since the patterns of the variations are different for different snRNAs, we used different strategies to extract reads mapped to these variable regions. In this manual, we only use uniquely mappable reads. You are free to explore the possibility of reassigning all reads mappable to snRNAs, based on the ratios inferred from uniquely mappable reads. However we don't think that it adds much value since it requires significant amount of unique reads for the determination of ratios reliable; and when there are large amounts of unique reads we do not care if the repetitive reads are to be added or not.

The input files are from bowtie mapping of RNA-seq reads, and the genome assembly is *D. melanogaster* Apr. 2006 (BDGP R5/dm3). The output of other mapping programs, including bowtie2, and wrappers that use bowtie/bowtie2 as the engines, have variations in the format, and therefore some of the commands for subsequent analysis need to be modified accordingly. For example: the labeling of mismatches is different for them. The following analysis procedure is also useful for other purposes, such as analysis of differential expression of the paralogs in different tissues or development. Since the following analysis is designed for 35nt RNA-seq reads, modifications are needed for some of them to work optimally for reads of different lengths. Please familiarize yourself with the basics of command line interface before attempting to use these commands. Note: the samtools retrieving reads by location from indexed BAM files is very efficient, and therefore the job submission command 'bsub' for 'samtools view' is not so necessary in most occasions.

Drosophila snRNA genes		
snRNA	Symbol	#GID
U1 5/7	snRNA:U1:21D	CR31656
	snRNA:U1:82Ea	
	snRNA:U1:82Eb	CR32862
	snRNA:U1:82Ec	
	snRNA:U1:95Ca	CR31341
	snRNA:U1:95Cb	CR32866
	snRNA:U1:95Cc	CR31185
U2 6/8	snRNA:U2:14B	CR32913
	snRNA:U2:34ABa	CR31850
	snRNA:U2:34ABb	CR31854
	snRNA:U2:34ABc	CR33788
	snRNA:U2:38ABa	CR32882
	snRNA:U2:38ABb	CR32878
	snRNA:U2:84Ca	
	snRNA:U2:84Cb	
U4	snRNA:U4:25F	CR32998
	snRNA:U4:38AB	CR32879
	snRNA:U4:39B	CR31625
U6	snRNA:U6:96Aa	CR31379
	snRNA:U6:96Ab	CR32867
	snRNA:U6:96Ac	CR31539
U5 7/8	snRNA:U5:14B	CR32914
	snRNA:U5:23D	CR32999
	snRNA:U5:34A	CR31853
	snRNA:U5:35D	CR32877
	snRNA:U5:38ABa	CR32881
	snRNA:U5:38ABb	CR32880
	snRNA:U5:39B	
	snRNA:U5:63BC	CR32908
U4atac	snRNA:U4atac:82E	CR32860
U6atac	snRNA:U6atac:29B	CR32989
U11	snRNA:U11:63F	CR34151
U12	snRNA:U12:73B	CR32162
U7	snRNA:U7	CR33504
LU	snRNA:LU	CR43708

Single copy snRNAs

Even though these snRNAs are all single copy genes (except that LU has an unexpressed pseudogene paralog, which takes away many reads from the expressed gene), we present the commands for retrieving RNA-seq reads mapped to them.

```
bsub samtools view -o Lu001.U4atac.sam Lu001 sorted.bam chr3R:1020726-1020885

bsub samtools view -o Lu001.U6atac.sam Lu001 sorted.bam chr2L:8389724-8389820

bsub samtools view -o Lu001.U7.sam Lu001 sorted.bam chr3L:3593823-3593893

bsub samtools view -o Lu001.U11.sam Lu001 sorted.bam chr3L:3893056-3893330

bsub samtools view -o Lu001.U12.sam Lu001 sorted.bam chr3L:16646869-16647106

bsub samtools view -o Lu001.LU.sam Lu001_sorted.bam chr2L:3046765-3046880
bsub samtools view -o Lu001.LUp.sam Lu001_sorted.bam chr2L:21644292-21644365
cat Lu001.LU.sam Lu001.LUp.sam > Lu001.LU.all.sam
awk '$14 == "NM:i:0"' Lu001.LU.all.sam > Lu001.LU.noMM.sam
rm Lu001.LU.sam Lu001.LUp.sam Lu001.LU.all.sam

wc -l Lu001.U4atac.sam Lu001.U6atac.sam Lu001.U7.sam Lu001.U11.sam Lu001.U12.sam Lu001.LU.all.sam
```

U1 snRNA

There are 5 U1 snRNAs in *Drosophila*: U1:21D, U1:82Eb, U1:95Ca, U1:95Cb and U1:95Cc. Three nucleotide variations at 70, 123 and 134 separate them into three different groups: U1:82Eb, U1:95Cc and U1:21D/U1:95Ca/U1:95Cb (see alignment of U1 paralogs below). The variable nucleotides at position 123 and 134 are close to each other and can be used to distinguish all three groups, therefore we searched for these fragments covering 123-134 in reads mapped to all U1 paralogs using grep or awk (grep is much faster than awk in general searching). Since the variable region used for analysis is only 12nt long, we cannot use the 'samtools view coordinate' method to retrieve reads covering this region. These 3 variant fragments are unique in U1 sequence and not anywhere else in the U1 gene. The following analysis of U1 snRNAs is not dependent on the size of the RNA-seq reads.

**U1 paralogs**	**strand**	**genomic locations**	**Fragments used**
U1:21D	-	chr2L:901491-901654	TGTAATTTTTGG
U1:82Eb	-	chr3R:773655-773818	TGTAATTTTTGT
U1:95Ca	-	chr3R:19685189-19685352	TGTAATTTTTGG
U1:95Cb	+	chr3R:19653592-19653755	TGTAATTTTTGG
U1:95Cc	+	chr3R:19652056-19652219	CGTAATTTTTGG

U1 snRNA paralog alignments:

```
U1 21D     ATACTTACCTGGCGTAGAGGTTAACCGTGATCACGAAGGCGGTTCCTCCGGAGTGAGGCTTGGCCATTGCACCTCGGCTGAGTTGACCTCTGCGATTATT 100
U1 95Ca    ATACTTACCTGGCGTAGAGGTTAACCGTGATCACGAAGGCGGTTCCTCCGGAGTGAGGCTTGGCCATTGCACCTCGGCTGAGTTGACCTCTGCGATTATT 100
U1 95Cb    ATACTTACCTGGCGTAGAGGTTAACCGTGATCACGAAGGCGGTTCCTCCGGAGTGAGGCTTGGCCATTGCACCTCGGCTGAGTTGACCTCTGCGATTATT 100
U1 95Cc    ATACTTACCTGGCGTAGAGGTTAACCGTGATCACGAAGGCGGTTCCTCCGGAGTGAGGCTTGGCCATTGCACCTCGGCTGAGTTGACCTCTGCGATTATT 100
U1 82Eb    ATACTTACCTGGCGTAGAGGTTAACCGTGATCACGAAGGCGGTTCCTCCGGAGTGAGGCTTGGCCATTGTACCTCGGCTGAGTTGACCTCTGCGATTATT 100
           ********************************************************************* ******************************
U1 21D     CCTAATGTGAATAACTCGTGCGTGTAATTTTTGGTAGCCGGGAATGGCGTTCGCGCCGTCCCGA 164
U1 95Ca    CCTAATGTGAATAACTCGTGCGTGTAATTTTTGGTAGCCGGGAATGGCGTTCGCGCCGTCCCGA 164
U1 95Cb    CCTAATGTGAATAACTCGTGCGTGTAATTTTTGGTAGCCGGGAATGGCGTTCGCGCCGTCCCGA 164
U1 95Cc    CCTAATGTGAATAACTCGTGCGCGTAATTTTTGGTAGCCGGGAATGGCGTTCGCGCCGTCCCGA 164
U1 82Eb    CCTAATGTGAATAACTCGTGCGTGTAATTTTTGTTAGCCGGGAATGGCGTTCGCGCCGTCCCGA 164
           ********************** ********** ******************************
```

Start from bowtie mapped bam files:

```
bsub samtools sort Lu001.bam Lu001 sorted
bsub samtools index Lu001 sorted.bam
```

Extract reads mapped to all five paralogs (optional, compare it to uniquely mappable reads):

```
bsub samtools view -o Lu001.U1 21D.sam Lu001 sorted.bam chr2L:901491-901654
bsub samtools view -o Lu001.U1 82Eb.sam Lu001 sorted.bam chr3R:773655-773818
bsub samtools view -o Lu001.U1 95Ca.sam Lu001 sorted.bam chr3R:19685189-19685352
bsub samtools view -o Lu001.U1 95Cb.sam Lu001 sorted.bam chr3R:19653592-19653755
bsub samtools view -o Lu001.U1 95Cc.sam Lu001 sorted.bam chr3R:19652056-19652219
```

Merge paralogs and remove mismatches:

```
cat Lu001.U1 * > Lu001.U1.sam
awk '$14 == "NM:i:0"' Lu001.U1.sam > Lu001.U1.noMM.sam
rm Lu001.U1 * Lu001.U1.sam
```

Extract reads covering the variable region by grepping:

```
grep CCAAAAATTACA Lu001.U1.noMM.sam > Lu001.U1 21D.U1 95Ca.U1 95Cb.sam
grep TGTAATTTTTGG Lu001.U1.noMM.sam >> Lu001.U1 21D.U1 95Ca.U1 95Cb.sam
grep ACAAAAATTACA Lu001.U1.noMM.sam > Lu001.U1 82Eb.sam
grep TGTAATTTTTGT Lu001.U1.noMM.sam >> Lu001.U1 82Eb.sam
grep CCAAAAATTACG Lu001.U1.noMM.sam > Lu001.U1 95Cc.sam
grep CGTAATTTTTGG Lu001.U1.noMM.sam >> Lu001.U1 95Cc.sam
```

Using bsub to submit these grep jobs (Note: grep with bsub produces a header of 30 lines that include the job description and log, make sure subtract these when counting the lines of output)

```
bsub -o Lu001.U1 21D.U1 95Ca.U1 95Cb1.sam grep CCAAAAATTACA Lu001.U1.noMM.sam
bsub -o Lu001.U1 21D.U1 95Ca.U1 95Cb2.sam grep TGTAATTTTTGG Lu001.U1.noMM.sam
bsub -o Lu001.U1 82Eb1.sam grep ACAAAAATTACA Lu001.U1.noMM.sam
bsub -o Lu001.U1 82Eb2.sam grep TGTAATTTTTGT Lu001.U1.noMM.sam
bsub -o Lu001.U1 95Cc1.sam grep CCAAAAATTACG Lu001.U1.noMM.sam
bsub -o Lu001.U1 95Cc2.sam grep CGTAATTTTTGG Lu001.U1.noMM.sam
```

U2 snRNA

Drosophila has 6 U2 paralogs: U2:14B, U2:34Aba, U2:34ABb, U2:34ABc, U2:38Aba and U2:38ABb. A total of 4 nucleotide variations separate them into 5 groups (see alignment of U2 paralogs). However these mismatches are scattered around the whole transcript, therefore we cannot use a single region to distinguish all of them.

Alignment of U2 paralogs

```
U2_38Aba   ATCGCTTCTCGGCCTTATGGCTAAGATCAAAGTGTAGTATCTGTTCTTATCAGCTTAACATCTGATAGTTCCTCCATTGGAGGACAACAAATGTTAAACT 100
U2_38ABb   ATCGCTTCTCGGCCTTATGGCTAAGATCAAAGTGTAGTATCTGTTCTTATCAGCT-AACATCTGATAGTTCCTCCATTGGAGGACAACAAATGTTAAACT 99
U2_14B     ATCGCTTCTCGGCCTTATGGCTAAGATCAAAGTGTAGTATCTGTTCTTATCAGCTTAACATCTGATAGTTCCTCCATTGGAGGACAACAAATGTTAAACT 100
U2_34ABc   ATCGCTTCTCGGCCTTATGGCTAAGATCAAAGTGTAGTATCTGTTCTTATCAGCTTAACATCTGATAGTTCCTCCATTGGAGGACAACAAATGTTAAACT 100
U2_34ABb   ATCGCTTCTCGGCCTTATGGCTAAGATCAAAGTGTAGTATCTGTTCTTATCAGCTTAACATCTGATAGTTCCTCCATTGGAGGACAACAAATGTTAAACT 100
U2_34ABa   ATCGCTTCTCGGCCTTATGGCTAAGATCAAAGTGTAGTATCTGTTCTTATCAGCTTAACATCTGATAGTTCCTCCATTGGAGGACAACAAATGTTAAACT 100
           ******************************************************* ********************************************
U2_38ABa   GATTTTTGGAATCAGACGGAGTGCTAGGGGCTTGCTCCACCTCTGTCACGGGTTGGCCCGGTATTGCAGTACCGCCGGGATTTCGGCCCAAC 192
U2_38ABb   GATTTTTGGAATCAGACGGAGTGCTAGGGGCTTGCTCCACCTCTGTCACGGGTTGGCCCGGTATTGCAGTACCGCCGGGATTTCGGCCCAAC 191
U2_14B     GATTTTTGGAATCAGACGGAGTGCTAGGGGCTTGCTCCACCTCTGTCACGGGTTGGCCCGGTATTGCAGTACCGCCGGGACTTCGGCCCAAC 192
U2_34ABc   GATTTTTGGAATCAGACGGAGTGCTAGGAGCTTGCTCCACCTCTGTCGCGGGTTGGCCCGGTATTGCAGTACCGCCGGGATTTCGGCCCAAC 192
U2_34ABb   GATTTTTGGAATCAGACGGAGTGCTAGGAGCTTGCTCCACCTCTGTCGCGGGTTGGCCCGGTATTGCAGTACCGCCGGGATTTCGGCCCAAC 192
U2_34ABa   GATTTTTGGAATCAGACGGAGTGCTAGGGGCTTGCTCCACCTCTGTCGCGGGTTGGCCCGGTATTGCAGTACCGCCGGGATTTCGGCCCAAC 192
           ****************************.******************.******************************** **********
```

In order to assign all U2 snRNA reads to the paralogs, we first determine the fraction each paralog takes, based on the 4 nucleotide variations. Assuming each paralog taking a fraction: *a*, *b*, *c*, *d*, *e* and *f*, we can establish the following system of 6 linear equations and solve each fraction:

Equations:

$a + b + c + d + e + f = 1$ (adds up to 1)

$c = d$ (34ABb and 34ABc are identical)

$f / (a + b + c + d + e) = r$ (measured ratios at variation 1)

$(a + e + f) : b : (c + d) / = x : y : z$ (measured ratios at variation 2 and 3)

$a / (b + c + d + e + f) = s$ (measured ratios at variation 4)

Solutions:

$a = s / (s + 1)$

$b = y / (x + y + z)$

$c = d = z / 2(x + y + z)$

$e = x / (x + y + z) - s / (s + 1) - r / (r + 1)$

$f = r / (r + 1)$

In case there are not enough reads mapped to the last variant nucleotide (e.g. in the embryonic stages RNA-seq using the SOLiD platform, modENCODE), we have to lump U2:14B and U3:38ABa (*a* and *e*) together, and treat them as equal. In fact, theses two isoforms are not very different in expression levels, as can be seen in the data. In this case, the solutions become:

$a = e = (x / (x + y + z) - r / (r + 1)) / 2$
$b = y / (x + y + z)$
$c = d = z / 2(x + y + z)$
$f = r / (r + 1)$

Since the distances among the 4 variable nucleotides are not all very big (73, 19 and 33), we have to determine the intervals that can be used to determine each ratio used in the equations. The 1st and last variants can be retrieved using 'samtools view coordinate', whereas the middle variants have to be retrieved using grep. Read lengths affect the variant regions used for read assignment to the 1st and last regions. Solutions to this problem for 35 nt reads are presented below:

U2 paralogs	location	strand	first variation (r, 35nt)	middle variants (m, 35nt)	last variation (s, 35nt)
U2:14B	chrX:16148705-16148896	+	chrX:16148760-16148760	GGCTTGCTCCACCTCTGTCA	chrX:16148887-16148887
U2:34ABa	chr2L:13211925-13212116	-	chr2L:13212062-13212062	GGCTTGCTCCACCTCTGTCG	chr2L:13211934-13211934
U2:34ABb	chr2L:13215839-13216030	+	chr2L:13215894-13215894	AGCTTGCTCCACCTCTGTCG	chr2L:13216021-13216021
U2:34ABc	chr2L:13244370-13244561	-	chr2L:13244507-13244507	AGCTTGCTCCACCTCTGTCG	chr2L:13244379-13244379
U2:38ABa	chr2L:19815614-19815805	-	chr2L:19815751-19815751	GGCTTGCTCCACCTCTGTCA	chr2L:19815623-19815623
U2:38ABb	chr2L:19812646-19812836	+	chr2L:19812701-19812701	GGCTTGCTCCACCTCTGTCA	chr2L:19812827-19812827

These are the commands used to extract reads covering these three regions and calculate the ratios to be used for determining the distribution of all reads (not just uniquely mappable reads).

Commands used to retrieve reads mapped to all U2 paralogs
```
bsub samtools view -o Lu001.U2 14B.sam Lu001 sorted.bam chrX:16148705-16148896
bsub samtools view -o Lu001.U2 34ABa.sam Lu001 sorted.bam chr2L:13211925-13212116
bsub samtools view -o Lu001.U2 34ABb.sam Lu001 sorted.bam chr2L:13215839-13216030
bsub samtools view -o Lu001.U2 34ABc.sam Lu001 sorted.bam chr2L:13244370-13244561
bsub samtools view -o Lu001.U2 38ABa.sam Lu001 sorted.bam chr2L:19815614-19815805
bsub samtools view -o Lu001.U2 38ABb.sam Lu001 sorted.bam chr2L:19812646-19812836
```

Merge paralogs and remove mismatches:
```
cat Lu001.U2 * > Lu001.U2.sam
awk '$14 == "NM:i:0"' Lu001.U2.sam > Lu001.U2.noMM.sam
rm Lu001.U2 * Lu001.U2.sam
```

Commands used to obtain the ratio r (by taking reads overlapping this nucleotide):
```
bsub samtools view -o Lu001.U2 r14B.sam Lu001 sorted.bam chrX:16148760-16148760
bsub samtools view -o Lu001.U2 r34ABa.sam Lu001 sorted.bam chr2L:13212062-13212062
bsub samtools view -o Lu001.U2 r34ABb.sam Lu001 sorted.bam chr2L:13215894-13215894
bsub samtools view -o Lu001.U2 r34ABc.sam Lu001 sorted.bam chr2L:13244507-13244507
bsub samtools view -o Lu001.U2 r38ABa.sam Lu001 sorted.bam chr2L:19815751-19815751
bsub samtools view -o Lu001.U2 r38ABb.sam Lu001 sorted.bam chr2L:19812701-19812701
```
Remove mismatches
```
awk '$14 == "NM:i:0"' Lu001.U2 r14B.sam > Lu001.U2 r14B.noMM.sam
awk '$14 == "NM:i:0"' Lu001.U2 r34ABa.sam > Lu001.U2 r34ABa.noMM.sam
awk '$14 == "NM:i:0"' Lu001.U2 r34ABb.sam > Lu001.U2 r34ABb.noMM.sam
awk '$14 == "NM:i:0"' Lu001.U2 r34ABc.sam > Lu001.U2 r34ABc.noMM.sam
awk '$14 == "NM:i:0"' Lu001.U2 r38ABa.sam > Lu001.U2 r38ABa.noMM.sam
awk '$14 == "NM:i:0"' Lu001.U2 r38ABb.sam > Lu001.U2 r38ABb.noMM.sam
```
Remove intermediate files
```
rm Lu001.U2 r14B.sam Lu001.U2 r34ABa.sam Lu001.U2 r34ABb.sam Lu001.U2 r34ABc.sam Lu001.U2 r38ABa.sam Lu001.U2 r38ABb.sam
wc -l *U2 r*
```
Commands used to obtain values x, y and z (by grepping the 20nt regions):
```
bsub samtools view -o Lu001.U2 14B.sam Lu001 sorted.bam chrX:16148705-16148896
bsub samtools view -o Lu001.U2 34ABa.sam Lu001 sorted.bam chr2L:13211925-13212116
bsub samtools view -o Lu001.U2 34ABb.sam Lu001 sorted.bam chr2L:13215839-13216030
bsub samtools view -o Lu001.U2 34ABc.sam Lu001 sorted.bam chr2L:13244370-13244561
bsub samtools view -o Lu001.U2 38ABa.sam Lu001 sorted.bam chr2L:19815614-19815805
bsub samtools view -o Lu001.U2 38ABb.sam Lu001 sorted.bam chr2L:19812646-19812836
cat Lu001.U2 14B.sam Lu001.U2 34ABa.sam Lu001.U2 34ABb.sam Lu001.U2 34ABc.sam Lu001.U2 38ABa.sam Lu001.U2 38ABb.sam > Lu001.U2.sam
awk '$14 == "NM:i:0"' Lu001.U2.sam > Lu001.U2.noMM.sam
rm Lu001.U2 14B.sam Lu001.U2 34ABa.sam Lu001.U2 34ABb.sam Lu001.U2 34ABc.sam Lu001.U2 38ABa.sam Lu001.U2 38ABb.sam Lu001.U2.sam
grep GGCTTGCTCCACCTCTGTCA Lu001.U2.noMM.sam > Lu001.U2 x.sam
grep TGACAGAGGTGGAGCAAGCC Lu001.U2.noMM.sam >> Lu001.U2 x.sam
grep GGCTTGCTCCACCTCTGTCG Lu001.U2.noMM.sam > Lu001.U2 y.sam
grep CGACAGAGGTGGAGCAAGCC Lu001.U2.noMM.sam >> Lu001.U2 y.sam
grep AGCTTGCTCCACCTCTGTCG Lu001.U2.noMM.sam > Lu001.U2 z.sam
grep CGACAGAGGTGGAGCAAGCT Lu001.U2.noMM.sam >> Lu001.U2 z.sam
```

Commands used to obtain ratios s (by taking reads overlapping this nucleotide, sparing the neighbor variants):
```
bsub samtools view -o Lu001.U2 s14B.sam Lu001 sorted.bam chrX:16148887-16148887
bsub samtools view -o Lu001.U2 s34ABa.sam Lu001 sorted.bam chr2L:13211934-13211934
bsub samtools view -o Lu001.U2 s34ABb.sam Lu001 sorted.bam chr2L:13216021-13216021
bsub samtools view -o Lu001.U2 s34ABc.sam Lu001 sorted.bam chr2L:13244379-13244379
bsub samtools view -o Lu001.U2 s38ABa.sam Lu001 sorted.bam chr2L:19815623-19815623
bsub samtools view -o Lu001.U2 s38ABb.sam Lu001 sorted.bam chr2L:19812827-19812827
```
Remove mismatches
```
awk '$14 == "NM:i:0"' Lu001.U2 s14B.sam > Lu001.U2 s14B.noMM.sam
awk '$14 == "NM:i:0"' Lu001.U2 s34ABa.sam > Lu001.U2 s34ABa.noMM.sam
awk '$14 == "NM:i:0"' Lu001.U2 s34ABb.sam > Lu001.U2 s34ABb.noMM.sam
awk '$14 == "NM:i:0"' Lu001.U2 s34ABc.sam > Lu001.U2 s34ABc.noMM.sam
awk '$14 == "NM:i:0"' Lu001.U2 s38ABa.sam > Lu001.U2 s38ABa.noMM.sam
awk '$14 == "NM:i:0"' Lu001.U2 s38ABb.sam > Lu001.U2 s38ABb.noMM.sam
```
Remove intermediate files
```
rm Lu001.U2 s14B.sam Lu001.U2 s34ABa.sam Lu001.U2 s34ABb.sam Lu001.U2 s34ABc.sam Lu001.U2 s38ABa.sam Lu001.U2 s38ABb.sam
```

U4 snRNA

Drosophila has 3 U4 paralogs: U4:25F, U4:38AB and U4:39B. Of these 3 paralogs, U4:25F has no fragments longer than 34 that are identical to the other 2 paralogs. The first 51 nucleotides are different among all of them and thus can be used to separate the 3 paralogs. To reassign the reads, first obtain the reads mapped to these three locations, then remove reads with mismatches, finally take reads that overlap the first 51 nucleotides. Read lengths do not affect the commands used for U4 snRNA paralogs.

U4 snRNA genomic coordinates of the variable regions:

U4 snRNAs	genomic coordinates	strand	variable region (35nt)
U4:25F	chr2L:5565619-5565766	+	chr2L:5565619-5565665

```
U4:38AB      chr2L:19810734-19810875   +            chr2L:19810734-19810779
U4:39B       chr2L:21215036-21215178   -            chr2L:21215133-21215178
```

U4 snRNA paralog alignment

```
U4 38AB    ATCTTTGCGCAGAGGCGATATCGTAACCAATGAAG-TTCTACTGAGGTGCGATTATTGCTAGTTGAAAACTTTAACCAATACCCCGCCATGGGGACGTGA 99
U4 39B     ATCTTTGCGCAGTGGCAATACCGTAACCAATGAAG-TCCTCCTGAGGTGCGGTTATTGCTAGTTGAAAACTTTAACCAATACCCCGCCATGGGGACGTGA 99
U4 25F     AACCTTGTGCAGTGGCAACATCGCAAGCAATGAAGTTCCAACTGAGCTGCGATTATTGCTAGTTGAAAACTAAAACCAATATCTCGCCCAGCGTAAG-GA 99
           *:* *** ****:***.* * ** ** ******** * *:.*****S****.******************::******** * ****.:* * *.* **
U4 38AB    AATACCGTC----CACTACGGCAATTTTTGGAAG-CCCGAGAGGGCCA- 142
U4 39B     AATACCGTC----CACTACGGCAATTTTTGGAAG-CCCGAGAGGGCTAA 143
U4 25F     TCTACGATCTTTAAGCTAAGGCAATTTTTTTAGGCCCCAAGTGGGCTGA 148
           :.*** .**    ..***.**********  *.* ***.**:**** .
```

Start from bowtie mapped bam files:

```
bsub samtools sort Lu001.bam Lu001 sorted
bsub samtools index Lu001 sorted.bam
```

Extract reads mapped to all U4 snRNA paralogs (optional, useful for comparing with reads mapped to only variable regions)

```
bsub samtools view -o Lu001.U4 25F.sam Lu001 sorted.bam chr2L:5565619-5565766
bsub samtools view -o Lu001.U4 38AB.sam Lu001 sorted.bam chr2L:19810734-19810875
bsub samtools view -o Lu001.U4 39B.sam Lu001 sorted.bam chr2L:21215036-21215178
```

Merge paralogs and remove mismatches:

```
cat Lu001.U4 * > Lu001.U4.sam
awk '$14 == "NM:i:0"' Lu001.U4.sam > Lu001.U4.noMM.sam
rm Lu001.U4 * Lu001.U4.sam
```

Extract reads mapped to variable regions:

```
bsub samtools view -o Lu001.U4_u25F.sam Lu001_sorted.bam chr2L:5565619-5565665
bsub samtools view -o Lu001.U4_u38AB.sam Lu001_sorted.bam chr2L:19810734-19810779
bsub samtools view -o Lu001.U4_u39B.sam Lu001_sorted.bam chr2L:21215133-21215178
```

Remove reads with mismatches:

```
awk '$14 == "NM:i:0"' Lu001.U4_u25F.sam >Lu001.U4_u25F.noMM.sam
awk '$14 == "NM:i:0"' Lu001.U4_u38AB.sam >Lu001.U4_u38AB.noMM.sam
awk '$14 == "NM:i:0"' Lu001.U4 u39B.sam >Lu001.U4 u39B.noMM.sam
```

Remove intermediate files:

```
rm Lu001.U4 u25F.sam Lu001.U4 u38AB.sam Lu001.U4 u39B.sam
```

U5 snRNA

Drosophila has 7 U5 paralogs: U5:14B, U5:23D, U5:34A, U5:35D, U5:38ABa, U5:ABb and U5:63BC. The 5' part of U5 is identical in all of them, but the 3' end is very different among them (see alignment of U5 paralogs). Reads spanning the 3' end variable region can be used to distinguish all of them from each other. In fact all reads must overlap the highlighted nucleotide ('s'), and this is enough for the retrieval of most if not all unique reads. There is no need for any adjustment of the coordinates for reads of different lengths.

U5 snRNA genomic coordinates of variable regions.

```
U5 snRNAs    genomic coordinates       strand       variable region
U5:14B       chrX:16148019-16148150    -            chrX:16148052-16148052
U5:23D       chr2L:3048701-3048831     +            chr2L:3048797-3048797
U5:34A       chr2L:13244848-13244974   +            chr2L:13244944-13244944
U5:35D       chr2L:15751557-15751682   -            chr2L:15751587-15751587
U5:38ABa     chr2L:19811948-19812074   -            chr2L:19811978-19811978
U5:38ABb     chr2L:19816414-19816540   +            chr2L:19816509-19816509
U5:63BC      chr3L:3090801-3090923     +            chr3L:3090895-3090895
```

U5 snRNA paralog alignment

```
U5 23D     ACTCTGGTTTCTCTTCAATTGTCGAATAAATCTTTCGCCTTTTACTAAAGATTTCCGTGGAGAGGAACACTCTAATGAGTCTAAACACAATTTTTGCTTA 100
U5 38ABb   ACTCTGGTTTCTCTTCAATTGTCGAATAAATCTTTCGCCTTTTACTAAAGATTTCCGTGGAGAGGAACACTCTAATGAGTCTAAACACAATTTTT--ATT 98
U5 38ABa   ACTCTGGTTTCTCTTCAATTGTCGAATAAATCTTTCGCCTTTTACTAAAGATTTCCGTGGAGAGGAACACTCTAATGAGTCTAAACTCAATTTTTG---T 97
U5 34A     ACTCTGGTTTCTCTTCAATTGTCGAATAAATCTTTCGCCTTTTACTAAAGATTTCCGTGGAGAGGAACACTCTAATGAGTCTAAAATAATCTTTTG---T 97
U5 35D     ACTCTGGTTTCTCTTCAATTGTCGAATAAATCTTTCGCCTTTTACTAAAGATTTCCGTGGAGAGGAACACTCTAATGAGTCTAAAATATTATTTTG---T 97
U5 14B     ACTCTGGTTTCTCTTCAATTGTCGAATAAATCTTTCGCCTTTTACTAAAGATTTCCGTGGAGAGGAACACTCTAATGAGTCTAAACACAATTTTTT-ATT 99
U5 63BC    ACTCTGGTTTCTCTTCAATTGTCGAATAAATCTTTCGCCTTTTACTAAAGATTTCCGTGGAGAGGAACACTCTAATGAGTCTAAA-ATAATTTTTA-GTA 98
           ************************************************************************************* : :: ****S   :
U5 34A     AGTG-CCCGGCGACTTCGGTAGC-----TGGG-CCA- 29
U5 35D     AGTG-CCCGGCGACTTTGGTAAC-----TGGG-CCA- 29
U5 63BC    -GTG-CCCTGTCGC----AAGAC-----TGGGGCCA- 25
U5 38ABa   -ATGACCTGGCTAAATATTTAGT-----TGGG-CCA- 29
U5 38ABb   -GAGGCCTGATAACTT--ATG-CT---ATCGGGCCA- 29
U5 14B     -GAGGCCTGATAACTT--ATG-TT---ATCGGGCCCA 30
U5 23D     -GAGCCCCGATGGCAT--TTGCCT---TTGGGGCCA- 30
              * **                     * ** **
```

Start from bowtie mapped bam files:

```
bsub samtools sort Lu001.bam Lu001 sorted
bsub samtools index Lu001 sorted.bam
```

Extract reads mapped to all U5 snRNA paralogs (optional, useful for comparing with reads mapped to only variable regions)

```
bsub samtools view -o Lu001.U5 14B.sam Lu001 sorted.bam chrX:16148041-16148150
bsub samtools view -o Lu001.U5 23D.sam Lu001 sorted.bam chr2L:3048701-3048831
bsub samtools view -o Lu001.U5 34A.sam Lu001 sorted.bam chr2L:13244848-13244974
bsub samtools view -o Lu001.U5 35D.sam Lu001 sorted.bam chr2L:15751557-15751682
bsub samtools view -o Lu001.U5 38ABa.sam Lu001 sorted.bam chr2L:19811948-19812074
bsub samtools view -o Lu001.U5 38ABb.sam Lu001 sorted.bam chr2L:19816414-19816540
bsub samtools view -o Lu001.U5 63BC.sam Lu001 sorted.bam chr3L:3090801-3090923
cat Lu001.U5 14B.sam Lu001.U5 23D.sam Lu001.U5 34A.sam Lu001.U5 35D.sam Lu001.U5 38ABa.sam Lu001.U5 38ABb.sam Lu001.U5 63BC.sam >
Lu001.U5.sam
```

Remove mismatches

```
awk '$14 == "NM:i:0"' Lu001.U5.sam > Lu001.U5.noMM.sam
```

Remove intermediate files

```
rm Lu001.U5 14B.sam Lu001.U5 23D.sam Lu001.U5 34A.sam Lu001.U5 35D.sam Lu001.U5 38ABa.sam Lu001.U5 38ABb.sam Lu001.U5 63BC.sam
Lu001.U5.sam
```

Extract reads mapped to the variable regions only

```
bsub samtools view -o Lu001.U5 u14B.sam Lu001 sorted.bam chrX:16148052-16148052
bsub samtools view -o Lu001.U5 u23D.sam Lu001 sorted.bam chr2L:3048797-3048797
bsub samtools view -o Lu001.U5 u34A.sam Lu001 sorted.bam chr2L:13244944-13244944
bsub samtools view -o Lu001.U5 u35D.sam Lu001 sorted.bam chr2L:15751587-15751587
bsub samtools view -o Lu001.U5 u38ABa.sam Lu001 sorted.bam chr2L:19811978-19811978
bsub samtools view -o Lu001.U5 u38ABb.sam Lu001 sorted.bam chr2L:19816509-19816509
bsub samtools view -o Lu001.U5 u63BC.sam Lu001 sorted.bam chr3L:3090895-3090895
```

Remove reads with mismatches

```
awk '$14 == "NM:i:0"' Lu001.U5 u14B.sam >Lu001.U5 u14B.noMM.sam
awk '$14 == "NM:i:0"' Lu001.U5_u23D.sam >Lu001.U5_u23D.noMM.sam
```

```
awk '$14 == "NM:i:0"' Lu001.U5 u34A.sam >Lu001.U5 u34A.noMM.sam
awk '$14 == "NM:i:0"' Lu001.U5 u35D.sam >Lu001.U5 u35D.noMM.sam
awk '$14 == "NM:i:0"' Lu001.U5 u38ABa.sam >Lu001.U5 u38ABa.noMM.sam
awk '$14 == "NM:i:0"' Lu001.U5 u38ABb.sam >Lu001.U5 u38ABb.noMM.sam
awk '$14 == "NM:i:0"' Lu001.U5 u63BC.sam >Lu001.U5 u63BC.noMM.sam
```

Remove intermediate files

```
rm Lu001.U5 u14B.sam Lu001.U5 u23D.sam Lu001.U5 u34A.sam Lu001.U5 u35D.sam Lu001.U5 u38ABa.sam Lu001.U5 u38ABb.sam Lu001.U5 u63BC.sam
```

U6 snRNA

Drosophila has 3 identical U6 paralogs: U6:96Ca, U6:96Cb and U6:96Cc. However, the Bowtie mapping procedure randomly assign reads to each location, therefore may create heterogeneity in the expression levels. Here we treat them as one gene, and use the total number of reads to analyze enrichment.

```
bsub samtools view -o Lu001.U6 96Ca.sam Lu001 sorted.bam chr3R:20381810-20381916
bsub samtools view -o Lu001.U6 96Cb.sam Lu001 sorted.bam chr3R:20382414-20382520
bsub samtools view -o Lu001.U6 96Cc.sam Lu001 sorted.bam chr3R:20382937-20383043
cat Lu001.U6 * > Lu001.U6.sam
awk '$14 == "NM:i:0"' Lu001.U6.sam > Lu001.U6.noMM.sam
rm Lu001.U6 * Lu001.U6.sam
```

Mouse snRNAs

Mammalian genomes usually contain multiple genes for each major spliceosomal snRNA. They are usually not well characterized due to their repetitive nature.

```
                   ...........(((((((((.(((((..........)))))))))))((((...(((.(((((...........))))).)))...)))).((((((((
mU1a1              ATACTTACCTGGCAGGGGAGATACCATGATCACGAAGGTGGTTTTCCCAGGGCGAGGCTTATCCATTGCACTCCGGA-TGTGCTGACCCCTGCGATTTCC 99
mU1a1v             ATACTTACCTGGCAGGGGAGATACCATGATCACGAAGGTGGTTTTCCCAGGGCGAGGCTCATCCATTGCACTCCGGA-TGTGCTGACCCCTGCGATTTCC 99
mU1b1b2            ATACTTACCTGGCAGGGGAGATACCATGATCATGAAGGTGGTTTTCCCAGGGCGAGGCTCACCCATTGCACTTTGGGGTGTGCTGACCCCTGCGATTTCC 100
mU1b6              ATACTTACCTGGCAGGGGAGATACCATGATCACGAAGGTGGTTTTCCCAGGGCGAGGCTCACCCATTGCACTTTGGGGTGTGCTGACCCCTGCGATTTCC 100
mU1b6v             ATACTTACCTGGCAGGGGAGATACCATGATCACGAAGGTGGTTTTCCCAGGGCGAGGCTCACCCATTGCACTTTGGGGTGTGCTGACCCCTGCGATTTCC 100
                   ******************************** ************************** * **********  **  **********************
                                                m                          n      r
                   (.......)))))).))).))))..............(((((((...(((....)))..)))))))
mU1a1              CCAAATGCGGGAAACTCGACTGCATAATTTGTGGTAGTGGGGG-ACTGCGTTCGCGCTCTCCCCTG 164
mU1a1v             CCAAATGCGGGAAACTCGACTGCATAATTTGTGGTAGTGGGGG-ACTGCGTTCGCGCTCTCCCCTG 164
mU1b1b2            CCAAATGCGGGAAACTCGACTGCATAATTTGTGGTAGTGGGGG-ACTGCGTTCGCGCTCTCCCCTG 165
mU1b6              CCAAATGCGGGAAACTCGACTGCATAATTTGTGGTAGTGGGGGAGCTGCGTTCGCGCGCTCCCCTG 166                TCCATTGCACTC
mU1b6v             CCAAATGTGGGAAACTTGACTGCATAATATGGGGTAGTGGGGG-GCTGCGTTCGCGCGCTCCCCTG 165                CCCATTGCACTT
                   ******* ******** *********** ** ***********  ************ ********
                          ------------------------
                         Separate mU1b6v from others (s)       t            u
```

Use the first 5 equations to solve the fractions, then use the last two to verify, because the last two equations are less reliable.
Note bowtie mapping prints out all possible mapping locations, while tophat randomly selects one mapped location if multiple locations are found.

```
a + b + c + d + e = 1
c/(a + b + d + e) = m
a/(b + c + d + e) = n
(a + b)/(c + d + e) = r
e/(a + b + c + d) = s
(a + b + c) : d : e = t1:t2:t3
(d + e)/(a + b + c) = u

a = n/(1+n)
b = 1 - (a + c + d + e)
c = m/(1+m)
d = 1/(r+1) - m(1+m) - s/(1+s)
e = s/(1+s)
```

```
U1_m
samtools view SRR192530.1245_only_sorted.bam mU1a1:33 | grep 'NM:i:0' | wc -l
samtools view SRR192530.1245_only_sorted.bam mU1a1v:33 | grep 'NM:i:0' | wc -l
samtools view SRR192530.1245_only_sorted.bam mU1b1b2:33 | grep 'NM:i:0' | wc -l
samtools view SRR192530.1245_only_sorted.bam mU1b6:33 | grep 'NM:i:0' | wc -l
samtools view SRR192530.1245_only_sorted.bam mU1b6v:33 | grep 'NM:i:0' | wc -l

samtools view SRR192530.1245_only_sorted.bam mU1b1b2:33 | grep 'NM:i:0' | wc -l
samtools view SRR192530.1245_only_sorted.bam mU1a1:33 > SRR192530.1245_only_sorted_m.sam
samtools view SRR192530.1245_only_sorted.bam mU1a1v:33 >> SRR192530.1245_only_sorted_m.sam
samtools view SRR192530.1245_only_sorted.bam mU1b6:33 >> SRR192530.1245_only_sorted_m.sam
samtools view SRR192530.1245_only_sorted.bam mU1b6v:33 >> SRR192530.1245_only_sorted_m.sam
cut -f1 SRR192530.1245_only_sorted_m.sam | sort -g | uniq | wc -l
rm SRR192530.1245_only_sorted_m.sam

U1_n
samtools view SRR192530.1245_only_sorted.bam mU1a1:60 | grep 'NM:i:0' | wc -l
samtools view SRR192530.1245_only_sorted.bam mU1a1v:60 | grep 'NM:i:0' | wc -l
samtools view SRR192530.1245_only_sorted.bam mU1b1b2:60 | grep 'NM:i:0' | wc -l
samtools view SRR192530.1245_only_sorted.bam mU1b6:60 | grep 'NM:i:0' | wc -l
samtools view SRR192530.1245_only_sorted.bam mU1b6v:60 | grep 'NM:i:0' | wc -l

samtools view SRR192530.1245_only_sorted.bam mU1a1:60 | grep 'NM:i:0' | wc -l
samtools view SRR192530.1245_only_sorted.bam mU1a1v:60 > SRR192530.1245_only_sorted_n.sam
samtools view SRR192530.1245_only_sorted.bam mU1b1b2:60 >> SRR192530.1245_only_sorted_n.sam
samtools view SRR192530.1245_only_sorted.bam mU1b6:60 >> SRR192530.1245_only_sorted_n.sam
samtools view SRR192530.1245_only_sorted.bam mU1b6v:60 >> SRR192530.1245_only_sorted_n.sam
cut -f1 SRR192530.1245_only_sorted_n.sam | sort -g | uniq | wc -l
rm SRR192530.1245_only_sorted_n.sam

U1_r
samtools view SRR192530.1245_only_sorted.bam mU1a1:62-78 | grep 'NM:i:0' | wc -l
samtools view SRR192530.1245_only_sorted.bam mU1a1v:62-78 | grep 'NM:i:0' | wc -l
samtools view SRR192530.1245_only_sorted.bam mU1b1b2:62-78 | grep 'NM:i:0' | wc -l
samtools view SRR192530.1245_only_sorted.bam mU1b6:62-78 | grep 'NM:i:0' | wc -l
samtools view SRR192530.1245_only_sorted.bam mU1b6v:62-78 | grep 'NM:i:0' | wc -l

samtools view SRR192530.1245_only_sorted.bam mU1a1:62-78 > SRR192530.1245_only_sorted_r1.sam
samtools view SRR192530.1245_only_sorted.bam mU1a1v:62-78 >> SRR192530.1245_only_sorted_r1.sam
cut -f1 SRR192530.1245_only_sorted_r1.sam | sort -g | uniq | wc -l

samtools view SRR192530.1245_only_sorted.bam mU1b1b2:62-78 > SRR192530.1245_only_sorted_r2.sam
samtools view SRR192530.1245_only_sorted.bam mU1b6:62-78 >> SRR192530.1245_only_sorted_r2.sam
samtools view SRR192530.1245_only_sorted.bam mU1b6v:62-78 >> SRR192530.1245_only_sorted_r2.sam
cut -f1 SRR192530.1245_only_sorted_r2.sam | sort -g | uniq | wc -l


U1_s
samtools view SRR192530.1245_only_sorted.bam mU1a1:107-131 | grep 'NM:i:0' | wc -l
samtools view SRR192530.1245_only_sorted.bam mU1a1v:107-131 | grep 'NM:i:0' | wc -l
samtools view SRR192530.1245_only_sorted.bam mU1b1b2:108-132 | grep 'NM:i:0' | wc -l
samtools view SRR192530.1245_only_sorted.bam mU1b6:108-132 | grep 'NM:i:0' | wc -l
samtools view SRR192530.1245_only_sorted.bam mU1b6v:108-132 | grep 'NM:i:0' | wc -l

samtools view SRR192530.1245_only_sorted.bam mU1a1:107-131 > SRR192530.1245_only_sorted_s.sam
samtools view SRR192530.1245_only_sorted.bam mU1a1v:107-131 >> SRR192530.1245_only_sorted_s.sam
samtools view SRR192530.1245_only_sorted.bam mU1b1b2:108-132 >> SRR192530.1245_only_sorted_s.sam
samtools view SRR192530.1245_only_sorted.bam mU1b6:108-132 >> SRR192530.1245_only_sorted_s.sam
cut -f1 SRR192530.1245_only_sorted_s.sam | sort -g | uniq | wc -l
samtools view SRR192530.1245_only_sorted.bam mU1b6v:108-132 | grep 'NM:i:0' | wc -l


U1_t
grep mU1 SRR192530.1245_only.sam > SRR192530.1245_only_U1.sam
grep GGACT SRR192530.1245_only_U1.sam | grep 'NM:i:0' | wc -l
grep GGAGC SRR192530.1245_only_U1.sam | grep 'NM:i:0' | wc -l
grep GGGCT SRR192530.1245_only_U1.sam | grep 'NM:i:0' | wc -l

U1_u
samtools view SRR192530.1245_only_sorted.bam mU1a1:156 | grep 'NM:i:0' | wc -l
```

```
samtools view SRR192530.1245 only sorted.bam mU1a1v:156 | grep 'NM:i:0' | wc -l
samtools view SRR192530.1245 only sorted.bam mU1b1b2:157 | grep 'NM:i:0' | wc -l
samtools view SRR192530.1245 only sorted.bam mU1b6:158 | grep 'NM:i:0' | wc -l
samtools view SRR192530.1245 only sorted.bam mU1b6v:157 | grep 'NM:i:0' | wc -l
```

```
mU2.1           ATCGCTTCTCGGCCTTTTGGCTAAGATCAAGTGTAGTATCTGTTCTTATCAGTTTAATATCTGATACGTCCTCTATCCGAGGACAATATATTAAATGGAT 100
mU2.2           ATCGCTTCTCGGCCTTTTGGCTAAGATCAAGTGTAGTATCTGTTCTTATCAGTTTAATATCTGATACGTCCTCTATCCGAGGACAATATATTAAATGGAT 100
mU2.4           ATCGCTTCTCGGCCTTTTGGCTAAGATCAAGTGTAGTATCTGTTCTTATCAGTTTAATATCTGATACGCCCTCTATCTGAGGACAATATATTAAATGGAT 100
mU2.5           ATCGCTTCTCGGCCTTTTGGCTAAGATCAAGTGTAGTATCTGTTCTTATCAGTTTAATATCTGATATGTCCTCTATCTGAGGACAATATATTAAATGGAT 100
                ****************************************************************** * ******** **********************

mU2.1           TTTTGGAACTAGGAGTTGGAATAGGAGCTTGCTCCGTCCACTCCACGCATCGACCTGGTATTGCAGTACCTCCAGGAACGGTGCAAC 187  CGTCCTCTATCC
mU2.2           TTTTGGAACTAGGAGTTGGAATAGGAGCTTGCTCCGTCCACTCCACGCATCGACCTGGTATTGCAGTACCTCCAGGAACGGTGCACC 187
mU2.4           TTTTGGAACTAGGAGTTGGAATAGGAGCTTGCTCCGTCCACTCCACGCATCGACCTGGTATTGCAGTACCTCCAGGAACGGTGCACC 187  CGCCCTCTATCT
mU2.5           TTTTGGGAATAGGAGTTGGAATAGGAGCTTGCTCCGTCCACTCCACGTATCAACCTGGTATTGCAGTACTTCCAGGAATGGTACACC 187  TGTCCTCTATCT
                ****** * ************************************** *** ***************** ******** *** ** *
```

mU2.2 and mU2.3 are identical. mU2.1 and mU2.2 only differs at nt186, thus not practical to distinguish. Therefore mU2.1, mU2.2 and mU2.3 are considered as one isoform. The first three nucleotide variations are used to separate them into three isoforms.

U2

```
samtools view SRR192530.1245_only_sorted.bam mU2.1 > SRR192530.1245_only_U2.sam
samtools view SRR192530.1245_only_sorted.bam mU2.2 >> SRR192530.1245_only_U2.sam
samtools view SRR192530.1245_only_sorted.bam mU2.3 >> SRR192530.1245_only_U2.sam
grep CGTCCTCTATCC SRR192530.1245_only_U2.sam | grep 'NM:i:0' | cut -f1 | sort -g | uniq | wc -l
samtools view SRR192530.1245_only_sorted.bam mU2.4 | grep CGCCCTCTATCT | grep 'NM:i:0' | wc -l
samtools view SRR192530.1245_only_sorted.bam mU2.5 | grep TGTCCTCTATCT | grep 'NM:i:0' | wc -l

samtools view SRR192530.1245_only_sorted.bam mU2.1:186-186 | grep 'NM:i:0' | wc -l
samtools view SRR192530.1245_only_sorted.bam mU2.2:186-186 | grep 'NM:i:0' | wc -l
samtools view SRR192530.1245_only_sorted.bam mU2.3:186-186 | grep 'NM:i:0' | wc -l
samtools view SRR192530.1245_only_sorted.bam mU2.4:186-186 | grep 'NM:i:0' | wc -l
samtools view SRR192530.1245_only_sorted.bam mU2.5:186-186 | grep 'NM:i:0' | wc -l
```

```
m_r_h_U4A       AGCTTTGCGCAGTGGCAGTATCGTAGCCAATGAGGTTTATCCGAGGCGCGATTATTGCTAATTGAAAACTTTTCCCAATACCCCGCCGTGACGACTTGCA 100
chicken_U4A     AGCTTTGCGCAGTGGCAGTATCGTAGCCAATGAGGTTAATCCGAGGCGCGATTATTGCTAATTGAAAACTTTTCCCAATACCCCGCCGTGACGACTTGCA 100
m_r_h_U4C       AGCTTTGCGCAGTGGCAGTATCGTAGCCAATGAGGTTTATCCGAGGCGCGATTATTGCTAATTGAAAACTTTTCCCAATACCCCGCCATGACGACTTGAA 100
chicken_U4C     AGCTTTGCGCAGTGGCAGTATCGTAGCCAATGAGGTTAATCCGAGGCGCGATTATTGCTAATTGAAAACTTTTCCCAATACCCCGCCATGACGACTTGAA 100
                ************************************* ************************************************* ********** *

all_m_r_h_c_U4  ATATAGTCGGCATTGGCAATTTTTGACAGTCTCTACGGAGACTGG 145
                *********************************************
```

U4

```
samtools view SRR018013.1245_only_sorted.bam mU4a:89-100 | grep 'NM:i:0' | wc -l
samtools view SRR018013.1245_only_sorted.bam mU4b:89-100 | grep 'NM:i:0' | wc -l
```

```
mU5.1           ACTCTGGTTTCTCTTCAGATCGTATAAATCTTTCGCCTTTTACTAAAGATTTCCGTGGAGAGGAACAACTCTGAGTCTTAAACCAATTTTTTGAGGCCTT 100
mU5.2           ACTCTGGTTTCTCTTCAGATCGTATAAATCTTTCGCCTTTTACTAAAGATTTCCGTGGAGAGGAACAACTCTGAGTCTTAAACCAATTTTTTGAGGCCTT 100
mU5.3           ACTCTGGTTTCTCTTCAGATCGTATAAATCTTTCGCCTTTTACTAAAGATTTCCGTGGAGAGGAACAATTCTGAGTCTTACACTAATTTTTTGAGGCCTT 100
mU5.4           ACTCTGGTTTCTCTTCAGATCGTATAAATCTTTCGCCTTTTACTAAAGATTTCCGTGGAGAGGAACAAATCTGAGTCTTAACCCAATTTTTTGAGGTCTT 100
mU5.5           ACTCTGGTTTCTCTTCAGATCGTACAAATCTTTCGCCTTTTACTAAAGATTTCCGTGGAGAGGAACATATCTGAGTCATTACC-AATTTTTTGAGGTCTT 99
mU5.6           ACTCTGGTTTCTCTTCAGATCGTATAAATCTTTCGCCTTTTACTAAAGATTTCCGTGGAGAGGAACAATTCTGAGTCTTAACCCAATTTTTTGAGGTCCT 100
                ************************ ******************************************  ******** *   * ************ * *
                                                                 Invariant loop                             Sm site
mU5.1           G-TTTCGGCAAGGCT 114   CTCTGAGTCTTAA
mU5.2           G-TCTTGACAAGGCT 114   CTCTGAGTCTTAA
mU5.3           G-CTTTAGCAAGGCT 114   TTCTGAGTCTTAC
mU5.4           G-TGCTTACAAGACT 114   ATCTGAGTCTTAA
mU5.5           GCTTCTTGCAAGGCT 114   ATCTGAGTCATTA
mU5.6           G-CTCGTGCAGGGCT 114   TTCTGAGTCTTAA
                *       ** * **
```

U5

```
samtools view SRR018013.1245_only_sorted.bam mU5.1:103-114 | grep 'NM:i:0' | wc -l
samtools view SRR018013.1245_only_sorted.bam mU5.2:103-114 | grep 'NM:i:0' | wc -l
samtools view SRR018013.1245_only_sorted.bam mU5.3:103-114 | grep 'NM:i:0' | wc -l
samtools view SRR018013.1245_only_sorted.bam mU5.4:103-114 | grep 'NM:i:0' | wc -l
samtools view SRR018013.1245_only_sorted.bam mU5.5:103-114 | grep 'NM:i:0' | wc -l
samtools view SRR018013.1245_only_sorted.bam mU5.6:103-114 | grep 'NM:i:0' | wc -l

samtools view SRR018013.1245_only_sorted.bam mU5.1 > SRR018013.1245_only_U5.12.sam
samtools view SRR018013.1245_only_sorted.bam mU5.2 >> SRR018013.1245_only_U5.12.sam
grep CTCTGAGTCTTAA SRR018013.1245_only_U5.12.sam | grep 'NM:i:0' | wc -l
samtools view SRR018013.1245_only_sorted.bam mU5.3 | grep TTCTGAGTCTTAC | grep 'NM:i:0' | wc -l
samtools view SRR018013.1245_only_sorted.bam mU5.4 | grep ATCTGAGTCTTAA | grep 'NM:i:0' | wc -l
samtools view SRR018013.1245_only_sorted.bam mU5.5 | grep ATCTGAGTCATTA | grep 'NM:i:0' | wc -l
samtools view SRR018013.1245 only sorted.bam mU5.6 | grep TTCTGAGTCTTAA | grep 'NM:i:0' | wc -l
```
